# A Study of Clinical, Microbiological, and Echocardiographic Profile of Patients of Infective Endocarditis

**DOI:** 10.1155/2014/340601

**Published:** 2014-11-04

**Authors:** Soumik Ghosh, Ratnakar Sahoo, Ranjit Kumar Nath, Nandini Duggal, Adesh Kumar Gadpayle

**Affiliations:** ^1^Department of Medicine, PGIMER, Dr. RML Hospital, New Delhi 110001, India; ^2^Department of Cardiology, PGIMER, Dr. RML Hospital, New Delhi 110001, India; ^3^Department of Microbiology, PGIMER, Dr. RML Hospital, New Delhi 110001, India

## Abstract

Infective endocarditis, a great masquerader, is a clinical entity which may present with a myriad of manifestations. Its changing epidemiological profile has been studied in the previous decades in both the developed and the developing nations. In this study, we strived to uphold the evolving clinical profile and its outcome from a government tertiary care hospital in Northern India. It was a descriptive, cross-sectional, observational study conducted over two years' period involving 44 patients diagnosed with definite infective endocarditis, according to modified Dukes' criteria. Demographic, clinical, microbiological, and echocardiographic data were analysed. Mean age of patients was 31 years. Rheumatic heart disease with regurgitant lesions was the commonest risk factor. Dyspnea and fever were the predominant symptom, and pallor and heart failure the commonest sign. Cultures were positive in 52% with *Staphylococcus,* the major isolate. Transesophageal echocardiography fared better than transthoracic one to define the vegetations. Mortality is reported in 4.5%. Prolonged duration of fever, pallor, hematuria, proteinuria, rheumatoid factor positivity, and large vegetations proved to be poor prognostic variables. Culture positive endocarditis, with persistent bacteremia, had higher incidence of acute renal failure. Right sided endocarditis was frequent in congenital lesions or IV drug user, whereas left sided endocarditis mostly presented with atrial fibrillation.

## 1. Introduction

Infective endocarditis (IE) is an infection of the cardiac valves or mural endocardium caused by bacteria and fungi producing a wide variety of systemic signs and symptoms through several mechanisms, including both sterile and infected emboli and various immunological phenomena. The modified Dukes' diagnostic criteria have been in use for case definition as definite or possible IE [1].

Despite recent advances in the field of cardiology and infectious disease medicine, IE remains a serious infection, with a stable incidence of* Streptococcus* spp. being the main causative agent. Of late, other pathogens have progressively emerged to be significant [2]. Culture-negative IE is also recognized as an important clinical entity [3].

Since in the Indian scenario very few studies [4, 5] have been undertaken on the changing epidemiological aspects of IE in the last decade and with the emergence of new advancements in the diagnostic modalities, we aim to investigate the clinical, microbiological, and echocardiographic profile of patients of IE reporting to a North Indian tertiary care hospital.

## 2. Material and Method

The study was a cross-sectional, observational study including 44 patients provisionally diagnosed as infective endocarditis according to definition proposed by modified Dukes' criteria over a period of 24 months from October 2011 to September 2013 presenting in the medical emergency or outpatient visit of Department of Medicine, PGIMER, Dr. Ram Manohar Lohia Hospital, New Delhi. Any patient presenting with signs or symptoms compatible with the diagnosis of IE as laid down in the Guidelines for the diagnosis and antibiotic treatment of endocarditis in adults, a report of the Working Party of the British Society for Antimicrobial Chemotherapy, 2012 [6], associated with “at risk” cardiac valve lesions [7], was considered for clinical evaluation and thus investigated. Any patient having documented treatment history of receiving antibiotics, oral or parenteral, within 7–10 days' duration for their febrile illness, was excluded from the study [6]. Pregnant women and patients less than 12 years of age and those not willing to give consent for the study were also excluded.

Detailed clinical history with past history of previous episodes of endocarditis, rheumatic heart disease (RHD), and previous treatment history was especially sought. Meticulous thorough physical examination was conducted with special emphasis on cardiac examination, any stigmata of IE, and its complication whatsoever. Investigations included a complete hemogram, routine serum chemistry profile (Table 2), a 12-lead electrocardiogram, chest X-ray PA view, urinalysis, screening for HIV, and serological tests for rheumatoid factor (RF), ANA, C-reactive protein (CRP), and ASO. Special investigation included microbiological analysis consisting of at least three sets of blood cultures, each drawn at least one hour apart from three different venipuncture sites, for aerobic media (BacT/ALERT) and fungal subcultures in Sabouraud's dextrose agar. BacT/ALERT PF culture bottles were used with the BacT/ALERT Microbial Detection System in qualitative procedures for enhanced recovery and detection of aerobic and facultative anaerobic microorganisms (bacteria and yeast) from blood.

All patients underwent a comprehensive transthoracic 2D-echocardiographic (TTE) analysis including two-dimensional, Doppler, and M-mode imaging on Philips HD11XE analyser with standardised view [8], by a single echocardiographer to minimise interobserver bias. The entire study was performed with special consideration to the anatomy and pathology of heart valves and intracardiac shunts, site, and number of vegetations, if any, size of the largest vegetation, nature of the valve vegetation along with special reference to left ventricular ejection fraction, any regional wall motion abnormalities, and abscesses or perforation sited at the involved valves and/or subvalvular apparatus, if any. In case of ambiguity or vegetations not properly visualized, it was further confirmed and corroborated with a transesophageal echocardiography (TEE) on Philips AW 21110A.

## 3. Results

In this study, a total of 44 patients in the age range of 14 to 61 years with the diagnosis of definite IE as per modified Dukes' criteria were analysed. However, the exact incidence of IE could not be calculated as population at risk was not defined. The mean age of patients was 31 years and male patients were more commonly encountered with a male : female sex ratio of about 3.4 : 1. The mean duration of hospitalization was 24 days. Majority of the patients were discharged after being conservatively treated for the episode of IE. Four patients (9%) are warranted for surgical intervention due to IE complicating the existing valvular heart disease. Two patients (4.5%) expired in the study (Table 1).

Analysis of major risk factors of the study patients revealed 64% having RHD, 23% having congenital heart disease (CHD), and 9% being intravenous drug user (IDU). However, all the cases were of native valve endocarditis. Out of the 4 patients with history of being IDU diagnosed with IE, all of them had tricuspid valve lesions and 2 patients had normal heart valves. Two patients were found to have nosocomial IE (4.5%): one due to placement of intravenous catheter for a prolonged period and the other due to a septic sequel of arthrotomy. Eight patients among all (18%) had a previous history of episode of IE too.

The study revealed 91% of the patients had fever as their chief complaints, majority of them being high grade with prolonged pyrexia (>2 weeks' duration). The mean duration of fever was estimated to be 19.4 days. All of the patients complained of some degree of dyspnea and they were divided according to NYHA grading: 50% presented with NYHA grade IV dyspnea, 41% with grade III, and the rest with grade II. New onset palpitation at rest was complained by 77% patients and 45% reported a significant weight loss.

General physical examination revealed 82% with pallor, 59% with clubbing, mostly grade II/III, 50% with pedal edema, and 14% with icterus. On clinical examination, 60% of the patients had tachycardia and 10 (23%) were in atrial fibrillation that was of new onset. Six patients (14%) presented with systemic hypotension due to cardiogenic shock. Splenomegaly was documented in 55% patients.

Examination of eyes and integuments revealed the following:

- subconjunctival hemorrhage in 6/44 (14% patients);
- optic disc hemorrhage in 9/44 (23% patients) and Roth spot in 2/44 (4.5% patients) by fundoscopic examination;
- Osler's nodes in 2/44 (4.5% patients);
- Janeway lesion in 9/44 (23% patients).

Regarding clinical outcomes, 50% of the patients presented with overt congestive cardiac failure with evidence of pulmonary edema. Eight patients (36%) had ARF with corresponding biochemical evidence of uremia (Table 3). Thromboembolic phenomena as a result of embolisation of vegetations of IE were encountered in twelve patients (27%): four of them had cerebral infarcts due to acute embolus resulting in hemiparesis, and remaining 8 patients had pulmonary embolism leading to infarcts and/or segmental collapse of lung parenchyma. Incidentally, all the 12 patients had large vegetations (size more than 10 mm in its longest axis); the former patients have left sided endocarditis, and the latter had vegetation on the right sided chambers, either being an IDU or having fungal etiology for IE.

**Figure 1:**
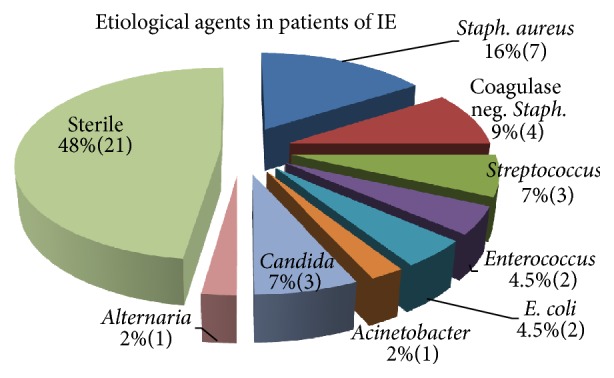
Pie chart to categorize the various microbial etiologies for IE on basis of blood cultures.

Fifty-two percent of the patients were blood culture positive for an etiological agent (Figure 1). Gram-positive organisms were mainly the offending pathogen amounting to 70% of the cases. Out of these* Staphylococcus aureus*, coagulase negative* Staphylococcus* (CONS),* Streptococcus* spp. (*mitis*,* bovis*, and* sanguis*), and* Enterococcus* were identified. Thirteen percent were Gram-negative pathogens:* E. coli* and* Acinetobacter *spp. isolated. The rest 17% were of fungal etiology: mostly* Candida* spp. with one of very unusual isolations of* Alternaria*, an uncommon pathogenic mould. Drug sensitivity reports obtained from bacterial cultures revealed that 2 out of 3* S. aureus* spp. and all CONS spp. were resistant to methicillin; and all* Streptococcus* spp. were also resistant to penicillin. Only a uniform susceptibility to Vancomycin was obtained for almost all bacterial isolates.

All the patients had elevated levels of C-reactive protein (CRP) on diagnosis of IE, with 50% of them having quantitative estimation of >32 mg/L and the rest had levels > 24 mg/L (normal reference range <4 mg/L). However, after treatment with antibiotic therapy repeat CRP showed undetectable levels. Due to immunologic interplay in sera in patients of IE, rheumatoid factor (RF) was tested and detected in 36% of the patients, without any evidence of active synovitis or periarticular swelling/effusion in individual patients. Serum for HIV I&II ELISA test were non-reactive for all the patients; but a false positive test was reported for HbsAg in only one patient, as he had no detectable anti-HbcAg antibody or HBV DNA titres.

**Figure 2:**
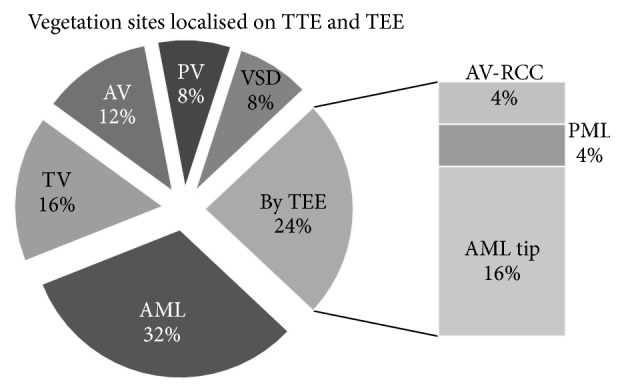
Segmental pie chart showing the localization of vegetation sites on various cardiac structures by TTE and TEE. Note: TTE failed to localise the vegetation of the mentioned sites determined by TEE. (AV: aortic valve, AML: anterior mitral leaflet, PV: pulmonary valve, PML: posterior mitral leaflet, RCC: right coronary cusp, and TV: tricuspid valve).

Vegetations in the cardiac structures or associated lesions, in each and every patient of IE, were either detected by transthoracic or confirmed by transesophageal echocardiography (Figure 2). Vegetations were localized on either damaged heart valves due to previous rheumatic activity or congenital cardiac lesions, and some were in normal structured cardiac valves with compromised function.

Among the patients with RHD (64%), most of them were found to be multivalvular and regurgitant lesions (Table 4). Mitral regurgitation (MR) was the most frequent valvular defect identified with incidence of 89% of all RHDs, followed by aortic regurgitation (AR), 68%, and tricuspid regurgitation (TR), 57%. Stenotic lesions that were also encountered were mitral stenosis (MS), 28%, and aortic stenosis (AS), 7%. Mitral valve prolapse (MVP) was seen in 14% of the patients.

**Figure 3:**
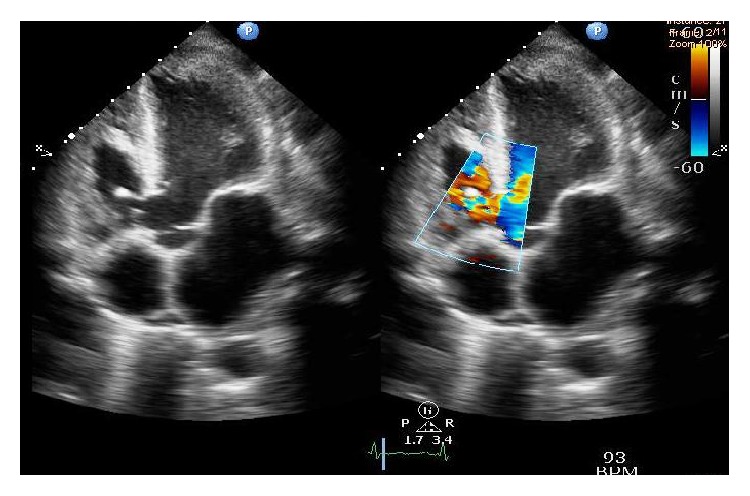
TTE showing vegetation attached to the lower margin of perimembranous VSD and papillary muscle of septal leaflet of tricuspid valve in one of our patients with CHD.

Among patients of IE with CHD comprising 23% of the study population, ventricular septal defect (VSD) was the predominant lesion (80%), associated with functional tricuspid regurgitation (Figure 3). We also had 2 patients of bicuspid aortic valve (BAV) disease (20%) associated with AS and AR. One patient also had pulmonic stenosis associated with VSD but no Fallot's physiology. Left ventricular ejection fraction (LVEF) being a reliable indicator of normal contractility of the heart was measured in each patient but only 14% of them recorded a decline of LVEF to less than 50% within the study population.

Statistical analysis using chi-square test and Fischer's Exact *t*-test analysis were calculated and significance was obtained when *P* value < 0.05 (Table 5). Patients of IE presenting with heart failure had a longer duration of fever (>15 days) (*P* = 0.001) and also manifested significant pallor (*P* = 0.005) and concomitant hematuria (*P* = 0.006), proteinuria (*P* = 0.004), and large vegetation size (*P* = 0.014). Patients presenting with acute renal failure as a complication of IE too had more hematuria (*P* = 0.012) and proteinuria (*P* = 0.001) and were with culture positive endocarditis with persistent bacteremia (*P* = 0.036). Embolic phenomena due to dislodgement of vegetation microthrombi were more associated with those patients who presented with evidence of prolonged pyrexia (*P* = 0.043), proteinuria (*P* = 0.027), positive reaction for RF (*P* = 0.04), large vegetation on echocardiography (*P* = 0.007), and risk factors for IE other than RHD (*P* = 0.013).

Analysis of echocardiographic findings revealed that the size of vegetation was directly proportionate to the duration of fever (*P* = 0.03) and positive reaction for RF (*P* = 0.02). Patients with rheumatic heart disease had relatively smaller vegetations than other risk factors (*P* = 0.05). Similarly, patients with right sided endocarditis had a relatively prolonged history of fever (*P* = 0.01) and most of them were associated with pallor (*P* = 0.037) relative to left sided endocarditis. Patients with right sided vegetations were frequently associated with congenital heart disease or normal valve with h/o being IDU (*P* = 0.001) and those with left sided vegetation in majority presented with atrial fibrillation (*P* = 0.009).

Multivariate regression analysis on the clinical variables, however, showed that patients presenting with heart failure had significant hematuria (*P* < 0.05), and those with acute renal failure had significant proteinuria (*P* = 0.03), rheumatoid factor positive (*P* = 0.021), and iron deficiency (*P* = 0.018); large vegetations on echocardiography had longer duration of fever (*P* = 0.025), and right sided lesions had significant hematuria (*P* = 0.036), proteinuria (*P* = 0.019), and larger vegetation size (*P* = 0.02) (Table 6).

## 4. Discussion

IE remains to be an uncommon disease with sporadic incidence, yet a serious entity in modern medicine, as its diagnosis requires a high degree of suspicion and treatment involves a holistic approach. Although there has been a notion that the incidence of IE has increased in recent years, contemporary population-based data have been lacking to support this opinion. Only a few studies involving profile of patients with IE were reported in the last decade in the region of Northern India [5, 9, 10].

In the present study, the mean age of patients was 31 years and male patients were more commonly encountered with a male : female sex ratio of about 3.4 : 1. This is comparable to other Indian studies done by Kothari et al., 2005 [9], Garg et al., 2005 [10], Subramanian et al., 2010, and Math et al., 2011, where the mean age was computed as 34 years, 27 years, 29 years, and 23.5 years, respectively. However, this is by far a contradistinction to the studies published in the West and the developed nations where the incidence of IE is higher among the age group of 5th to 6th decade of life. This is attributed to the fact that in Indian scenario, RHD is still the most prevalent risk factor for IE which makes the younger population vulnerable to infection of the diseased heart valves. Moreover, the incidence of untreated or undiagnosed CHD IDU in the young is also a major factor. As in recent studies from abroad, done by Weinberger et al. [11], implicating mitral valve prolapse [12, 13] as an important underlying cardiac valve lesion, our study only had 4 patients (9%) associated with IE.

**Figure 4:**
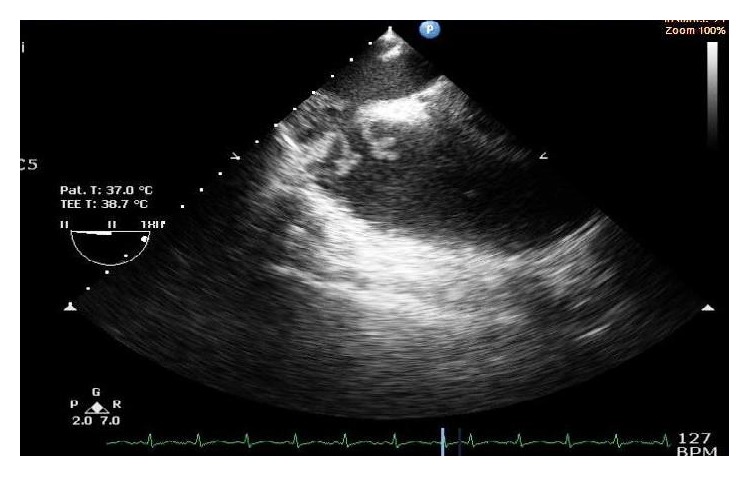
TEE showing large vegetations attached to the anterior and septal leaflet of a normal tricuspid valve in one of our IDU patients presenting with IE.

We encountered only native valve endocarditis in our study population, which may be due to the fact that either postoperative prosthetic valve patients followed a strict follow-up with adequate medical care or patients with predisposing cardiac conditions were previously unaware of or lack surgical prosthetic management in this region. Four patients with history of being IDU (9%) were diagnosed with IE (Figure 4), the incidence of which is as previously studied by Miro et al. [14].

Regarding the physical examination, pallor was the commonest sign (82%); however anemia was evident in only 68% patients. Patients with iron deficiency state, determined by serum iron concentration and transferrin saturation, were almost comparable with those having anemia. However, microcytosis constituted half of the population with anemia indicating severe iron deficiency state only, due to previous poor nutritional state probably. The rest had normocytic anemia reflecting anemia of chronic inflammation. Incidentally, multivariate regression analysis has shown significant association of iron deficiency in patients with history of prolonged duration of fever. Thus, the relation of anemia of chronic inflammation with iron deficiency state in IE patients invites further study [15]. Icterus was present in 8 patients, mainly due to sepsis (4 patients), congestive heart failure (2 patients), or both (2 patients). The other clinical signs obtained were similar to the frequency distribution of population studied by identical series [5, 10].

While comparing the culture positivity of cases of IE, it is found that definitive microbial etiology is underreported in Indian studies compared to the Western literature, as culture positivity was reported as 23%, 41%, and 67% by Subramanian et al., Math et al., and Garg et al., respectively; however, the European heart survey [16] reported that 86% of their patients were with culture positive endocarditis. Blood culture negative endocarditis (BCNE) cases are now less prevalent nowadays, since a myriad of fastidious organisms are identified with the help of serological and molecular diagnostic advancements like the* Brucella*,* Bartonella*,* Coxiella*,* Tropheryma*,* Legionella*,* Mycoplasma*, and so forth. Few case reports and a recent prospective study on large number of BCNE cases highlight the zoonotic link of IE with the causative organism [17, 18]. Incidentally, in our study we had 48% BCNE cases, relying on standard culture techniques; but majority of the patients with BCNE (12 of 21) epidemiologically were either housewives or farmers by occupation highlighting their agrarian background in Indian setup.

A mixed bag of opinion is available regarding the etiological agents for IE, while reviewing previously established studies globally, on temporal trends of the disease. Referring to the Indian studies, Garg et al. 2005 documented higher prevalence of* Streptococcus* (23%) for the most frequent etiological agent. Math et al. 2011 reported an equal incidence of* Staphylococcus* and* Streptococcus* etiology with Gram-positive organisms predominating. Again Subramanian et al. 2010 reiterated the importance of* Streptococcus viridians* reporting an incidence of 55% among all culture positive growths. In studies from abroad, a 10-year multicentric study in US by Bor et al. [19], 1999–2008, reported incidence of* Staphylococcus* and* Streptococcus* in cultures to be 28% and 24%, respectively. Rostagno et al. [20] published a study with higher incidence of* Staphylococcus* spp. (40%) than Streptococcal family (29%) in 2010. A 5-year prospective study in Olmsted County, 2010, reported an equal prevalence of either organisms, 12% each, with a significant increase in frequency of coagulase negative* Staphylococcus* epidemiology, 8%. Similar results of equal incidence of the two Gram-positive cocci were reported by Fedeli et al. [21] in 2011 in a record linkage analysis data of 1863 patients of IE. In an international collaborative prospective cohort study in over 25 countries, Murdoch et al. [22] showed the emerging significance of* Staphylococcus* as the predominant organism for IE, except in South America where* Streptococcus* etiology due to higher prevalence of RHD was found and significant proportion of cases due to fastidious organisms reported from European countries. Castillo et al., [23] too, reported a significant increase in causal epidemiology for* Staphylococcus* etiology in 2011. In contradistinction a maintained higher incidence of* Streptococcus* bacteremia in IE was established by Gotsman et al. 2007 [24] and Sucu et al. [25] and Takayama et al. [26] in 2010. In this study, although RHD proved to be a major cardiac substrate at risk, Staphylococcal cultures were the majority etiology, reiterating the paradigm shift of causative pathogen involving IE. Drug sensitivity reports obtained from bacterial cultures revealed a uniform susceptibility of almost all organisms to Vancomycin, which is still considered a potent bactericidal agent in cases of Enterobacteriaceae family and methicillin resistant* Staphylococcus aureus* (MRSA) bacteremia.

In the present study, RHD has been found to be the predominant cardiac condition among the risk factors for IE (64%) followed by CHD in adults which is almost one-third of the incidence (23%). Among similar Indian series elaborating on the predisposing factors for IE, Choudhury et al. [27] reported an incidence of 42% RHD and 33% CHD in his study, Garg et al. 47% and 29%, and Subramanian et al. 70% and 26%, respectively, in their studies. This nonuniformity most probably depicts the heterogeneity of the risk factors and presenting population of IE pertaining to their region of study. Incidentally, the prevalence of RHD in India, as documented by various population-based studies in the recent decade, is 5.8–9.7/1000 population [28, 29], which arguably is one of the highest in the world. Concurrently, the presence of uncorrected CHD in children less than 15 years in India has been speculated in few studies as 4.2/1000 population [30], but no systematic data on prevalence among adults was available.

Left sided endocarditis predominated in our study (66%) due to higher prevalence of RHD as a major risk factor and mitral and aortic valves being more vulnerable to deforming effect of rheumatic activity. Vegetations found in the right sided chambers of the heart (33%) were most commonly associated with CHD, that is, VSD involving the right ventricular aspect of septal defect forming the low pressure zone and the associated septal cusp of tricuspid leaflet; and the rest one-third were cases of being IDU where vegetations were seen implanted on tricuspid leaflets of anatomical normal tricuspid valve resulting in functional regurgitation. However, the various valve apparatus and perivalvular complications that were evidenced in routine echocardiography, like valve leaflet prolapse or perforation, chordal rupture, or annular abscess, were all associated with left sided chamber endocarditis.

Seventy-six percent of all vegetations were primarily detected by transthoracic echocardiography (TTE). Most of the vegetations diagnosed on TTE were large sized and importantly all right sided endocarditis were detectable with the help of TTE. The rest were diagnosed on transesophageal echocardiography (TEE); remarkably all of them were left sided endocarditis: located on either anterior or posterior mitral leaflet or tip or right coronary cusp of aortic valve, that is, the structures which are located more posteriorly in relation to precordium (Figure 2). This confers with the better degree of sensitivity (76–100%) and specificity (94%) of TEE over TTE (65% and 76%, resp.) for identifying vegetations and perivalvular complication in both adults and children [31, 32]. According to the recommendations, TTE is considered the first-line diagnostic modality in suspected IE; however TEE should always be sought in cases of highly suspicious IE where TTE is normal [33].

Vegetation size was also accurately recorded in each and every patient and a size of more than or equal to 10 mm in its longest axis was considered large vegetation. Size of vegetation did bear a significant prognosis in all patients of IE as previously studied by Gotsman et al., 2007 [24], influencing the clinical outcome of the patients and as a measure of response to treatment and need for surgical intervention. Incidentally in our study, patients with large sized vegetation had longer duration of fever at presentation, increased incidence of heart failure, and embolic phenomena and were directly proportionate to RF positivity. Similarly patients with congenital cardiac lesions or IDU patients with normal valves had larger vegetations than their RHD counterparts.

Surgical opinion was sought in all the cases of IE studied but only 4 patients (all with RHD with preexisting valvular deformity with clinical deterioration) could be operated on. Though surgical indication was present in other patients, it was not feasible due to either lack of consent for operative procedure or economic constraints of the patients' part. There was no mortality in those managed surgically and who had a better outcome as also reported by Gupta et al. [34] Patients with cardiogenic shock were advised to continue inotropic support in order to stabilise preoperatively, but two of them succumbed within a couple of days' admission.

During statistical computation using multivariate regression analysis, certain unprecedented associations were observed. Hematuria was significantly associated with patients presenting with heart failure and right sided endocarditis. This invites consideration which was possibility of cardiorenal syndrome as there was also significant proteinuria associated, but systemic renal embolism can be ruled out in cases of vegetations involving right sided chambers. Patients developing acute renal failure were frequently found to be RF positive and iron deficient, the significance of which is undetermined and needs further deliberation. In contrast, large sized vegetation had expectedly significant longer history of fever at presentation but was localised commonly on the right side, which differs from previous reports [35, 36].

The following were the limitations of this study.

1. All patients diagnosed as IE were included in the study, irrespective of the status of primary or referral case.
2. Pediatric age group <12 years was excluded thus affecting the predisposing patients at risk due to CHD lesions.
3. Not a single case of endocarditis of prosthetic valve was obtained in the study.
4. Latest and sophisticated serological investigations for identification of fastidious organisms could not be included.

## 5. Conclusion

Infective endocarditis remains a constant source of menace in medical practice, with associated morbidity and mortality. RHD continues to be the commonest risk factor for IE due to increased predisposition of deformed malfunctioning heart valves in RHD. Younger age group is predominantly seen to be predisposed with mean age group in thirties. Fever, dyspnea, and palpitation are the commonest symptom, and pallor, clubbing, presence of regurgitant murmur, and evidence of heart failure were the frequent signs encountered. Vegetations are considered the cardinal lesion in echocardiography associated with its complications with TEE faring slightly better than TTE in detection of lesions. Rheumatic mitral regurgitation was the commonest valvular lesion, with anterior mitral leaflet being the commonly occurring site of localizing of vegetation. Size of the vegetations appears to be an independent factor for risk of embolic phenomena in patients suffering from IE. Blood cultures grown are positive in only half of the cases, with* Staphylococcus aureus* and coagulase negative* Staphylococcus* being obtained in a majority of them. Vancomycin still emerges as a dependent antibiotic susceptible to the varied bacterial flora grown on blood culture and sensitivity testing. Rheumatoid factor proves to be an important surrogate marker for underlying immunological phenomena due to persistent bacteremia.

## Figures and Tables

**Table 1:** Summary of clinical profile of patients of IE (*n* = 44).

Parameters	Mean ± SD	Percentage
Age	31 ± 11.6 years	—
Sex	34 males/10 females	M : F:: 3.4 : 1
Duration of hospitalisation	24 ± 8.6 days	—

Fever	40	91%
Duration	19 ± 7.9 days	—
Dyspnea		
NYHA grade		
IV	22	50%
III	18	41%
II	4	9%
Palpitation (sudden onset)	34	77%
Weight loss	20	45%

Pallor	36	82%
Clubbing	26	59%
Pedal edema	22	50%
Icterus	6	14%
Raised JVP	22	50%
AF (new onset)	10	23%
Hepatomegaly	16	36%
Splenomegaly	24	55%
Cardiogenic shock	6	14%
	(RHD: 4/CHD: 2)	

Clinical outcomes		
Heart failure	22	50%
Acute renal failure	16	36%
Embolic events		27%
Pulmonary	8	
Cerebral	4	

Prognosis		
Discharge	42	95.5%
Death	2	4.5%

**Table 2:** Baseline laboratory investigation values.

Parameters	Mean ± SD	Ref. range
Hemoglobin (gm%)	9.6 ± 1.04	12–15
TLC	11.9 ± 4.98 × 10^3^	4–11 × 10^3^
ESR (mm 1st hr)	25 ± 13.5	<10
Neutrophilia	79% ± 10.1%	<65%
MCV (fL)	84 ± 5	80–96
Blood urea (mg%)	60 ± 60.4	15–40
Iron saturation	17.1 ± 17.9	20–50
Bilirubin (mg%)	1.04 ± 1.07	0.2–1.0
SGOT (IU/L)	55 ± 58.5	15–45
SGPT (IU/L)	40 ± 39.8	15–45
Albumin (gm%)	3.3 ± 0.7	3.5–4.5
Creatinine (mg%)	1.77 ± 2.8	0.5–1.5

TLC = total leucocyte count; ESR = erythrocyte sedimentation rate; MCV = mean corpuscular volume; SGOT = serum glutamate oxaloacetate transferase; SGPT = serum glutamate pyruvate transferase.

**Table 3:** Incidence of important clinicopathological conditions.

Variables^*^	Percentage
Proteinuria	68%
Hematuria	50%
Anemia	68%
Iron def.	59%
Microcytosis	32%
Uremia	36%
Jaundice	18%
Hypoalbuminemia	27%
Neutrophilia	72%

^*^Attributes: proteinuria = spot urinary protein >30 mg/dL; hematuria = >3 RBCs/hpf in urine; anemia = Hb <12 gm%; iron deficiency = transferrin saturation <20%; microcytosis = MCV < 80 fL; uremia = blood urea >45 mg%/serum creatinine >1.5 mg%; jaundice = bilirubin >2.5 mg%; hypoalbuminemia <3 gm%; neutrophilia = TLC > 11 × 10^3^/*μ*L.

**Table 4:** Valvular lesions associated with IE.

Rheumatic heart disease	28 (64%)	Congenital heart disease	10 (22%)

Mitral stenosis	8	Ventricular septal defect	8
Mitral regurgitation	25	With TR	4
Aortic stenosis	2	With PS	1
Aortic regurgitation	19	Bicuspid aortic valve	2
Tricuspid regurgitation	16	With AS	1
Mitral valve prolapse	4	With AR	2

Marfan's syndrome	2 (5%)	Normal valve	4 (9%)

Aortic regurgitation (post-AVR)	1	Tricuspid regurgitation	4
Mitral regurgitation	2	Mitral regurgitation	2

**Table 5:** Correlation between clinical, echocardiographic, and microbiological variables with statistical significance.

Variables	Heart failure	ARF	Embolic events	Vegetation size	Vegetation site
Duration of fever	**0.001**	0.155	**0.043**	**0.030**	**0.010**
Fever grade	**0.039**	0.170	0.459	**—**	**—**
Pallor	**0.005**	0.170	0.402	0.433	**0.037**
Hematuria	**0.006**	**0.012**	0.199	0.223	0.059
Proteinuria	**0.004**	**0.001**	**0.027**	**—**	**—**
Risk factors	0.063	0.751	**0.013**	**0.005**	**0.001**
ECG	0.472	0.237	0.697	0.148	**0.009**
RF	0.086	0.067	**0.040**	**0.002**	0.751
Cultures	0.349	**0.036**	0.194	**—**	**—**
Iron saturation	1.000	0.093	1.000	**—**	**—**
Vegetation size	**0.014**	0.164	**0.007**	NA	—
Vegetation site	**0.001**	0.468	**0.001**	**—**	NA

Calculated by Pearson's chi-square test and Fischer's Exact test analysis and significance obtained (highlighted in bold) when *P* value < 0.05.

Attributes:

duration of fever: presence of prolonged pyrexia (>14 days' duration); fever grade: high grade fever >101°F; risk factors: presence of predisposing cardiac condition like CHD or being IDU apart from RHD; ECG: presence of tachy/bradyarrhythmias; RF: rheumatoid factor positive; cultures: blood cultures positive compatible to modified Duke's criteria; iron saturation; presence of iron deficiency; vegetation size: vegetations >10 mm size in their long axis; vegetation site: right sided chamber/valvular lesions.

**Table 6:** Multivariate regression analysis of disease outcome with various clinical variables indicating *P* value for significance.

Outcomes	Duration of fever	Hematuria	Proteinuria	Rheumatoid factor	Iron deficiency	Vegetation size
Heart failure		**0.048**				
Acute renal failure			**0.03**	**0.021**	**0.018**	
Vegetation size	**0.025**					
Vegetation site		**0.036**	**0.019**			**0.02**
